# Alveolar bone grafting

**DOI:** 10.4103/0970-0358.57200

**Published:** 2009-10

**Authors:** Jan Lilja

**Affiliations:** Department of Plastic Surgery, Sahlgrenska University Hospital, Göthenburg, Sweden

**Keywords:** Bone grafting, Alveolar bone grafting, Management of bony cleft

## Abstract

In patients with cleft lip and palate, bone grafting in the mixed dentition in the residual alveolar cleft has become a well-established procedure. The main advantages can be summarised as follows: stabilisation of the maxillary arch; facilitation of eruption of the canine and sometimes facilitation of the lateral incisor eruption; providing bony support to the teeth adjacent to the cleft; raising the alar base of the nose; facilitation of closure of an oro-nasal fistula; making it possible to insert a titanium fixture in the grafted site and to obtain favourable periodontal conditions of the teeth within and adjacent to the cleft. The timing of the ABG surgery take into consideration not only eruption of the canine but also that of the lateral incisor, if present. The best time for bone grafting surgery is when a thin shell of bone still covers the soon erupting lateral incisor or canine tooth close to the cleft.

## HISTORICAL BACKGROUND

Von Eiselsberg (1901) and Lexer (1908) were the first to use autogenous bone to graft the cleft maxilla by a free bone or pedicled soft tissue and bone of the little finger. Drachter (1914) was the first to report the closure of a cleft with tibial bone and periosteum.

## PRIMARY BONE GRAFTING

Primary and early secondary bone grafting were practised mainly in the 1950s and 1960s by a whole generation of cleft surgeons.[1] The indication for primary bone grafting was- elimination of bone deficiency, stabilization of the pre-maxilla, creation of new bone matrix for eruption of teeth in the cleft area and augmentation of the alar base. There were also expectations of normalization or even stimulation of maxillary growth.[2]

Since 1964 many publications have been suggesting that grafting at this early stage causes serious growth disturbances of the middle third of the facial skeleton.[3,4] The operative technique that involves the vomero-premaxillary suture was found to cause inhibition of maxillary growth.[5] Though a few centres still perform the early bone grafting procedure it was abandoned in most cleft lip and palate centres worldwide.

## SECONDARY BONE GRAFTING

Secondary bone grafting, meaning bone grafting in the mixed dentition, became an established procedure after abandoning primary bone grafting. The pre-requisites were precise timing, operating technique, and sufficiently vascularized soft tissue. The advantages of primary bone grafting allowing tooth eruption through the grafted bone could also be maintained. Furthermore, secondary bone grafting can stabilize the dental maxillary arch, improving the conditions for prosthodontic treatment such as crowns, bridges and implants. It will also facilitate eruption of teeth increasing the amount of bony tissue on the alveolar crest allowing orthodontic treatment. Bony support to teeth neighbouring the cleft is a pre-requisite for orthodontic closure of the teeth in the cleft region. Thereby more favourable hygienic conditions will be achieved reducing caries and periodontal inflammation. Speech problems caused by irregular positioning of articulators, or escape of air through the oronasal communication may also be improved. Secondary bone grafting can also be used to augment the alar base of the nose to achieve symmetry with the non-cleft side, thereby improving facial appearance.

## LATE SECONDARY BONE GRAFTING

Bone grafting has a lower success rate when performed after canine eruption compared to before the eruption. It has also been found that the possibility for orthodontic closure of the cleft in the dental arch is smaller than in patients grafted before canine eruption. In adults oral hygiene is of major importance. The surgical procedure should include drilling of multiple small openings through the cortical layer into the cancellous layer facilitating in growth of blood vessels into the graft.

## CHOICE OF BONE GRAFTING MATERIAL FOR ALVEOLUS

Survival of the donor tissue is an important aspect in bone grafting. Under optimal conditions, the osteogenic cells survive the surgical procedure. Johanson and Röckert (1961) proved in histological and micro-radiographical clinical and experimental studies, that cancellous autogenous bone grafts, harvested from either tibia or iliac crest, were transformed to the same structure as the surrounding palate. After six months it was not possible, micro-radiographically or morphologically, to distinguish a biopsy sample from the graft region from one taken from a normal palate at the same age. Furthermore, the architecture of the graft appears to adapt to the functional requirements.

### Cancellous bone

The formation of new bone starts on the surface of the pre-existing trabeculae. Cancellous bone is more vascular, has more space, contains more bone regeneration and has better ingrowth of new bone from the adjacent bone segments. In principle, cancellous autografts heal primarily by osteogenesis, followed considerably later by resorption of the bone trabeculae in the transferred donor tissue.[6]

### Cortical bone

Early establishment of nutrition to cortical bone cells requires restoration of flow through existing vessels or canaliculi and ingrowth of capillaries. A cortical graft will usually die and be replaced by invasion of bone cells originating from the recipient site.[6] The metabolic turnover and remodelling/transformation of cortical bone are much slower than in cancellous bone, making re-establishment of the tooth-bearing function of the alveolar process in the cortical graft unfeasible.

## SOFT TISSUE COVERAGE

Boyne and Sands 1972, and Åbyholm *et al.* 1981 were the first to stress on the importance of flap design with the gingival mucoperiosteal flaps in secondary bone grafting to maxillary clefts. Histologically gingival/masticatory mucosa consists of a layer of keratinized stratified squamous epithelium and dense and firm lamina propria with immovable attachments to underlying teeth and bone. The gingiva, therefore, is a suitable surface to support the masticatory load and protect against chemical and bacterial damage. The gingival muco-periosteal flaps have a broad base and excellent vascularity and provide, after adequate mobilization, a tension-free closure.[7]

## DONOR SITES FOR HARVESTING BONE GRAFTS

Various donor sites have been used for harvesting bone grafts. Autogenous cancellous bone from anterior iliac crest is used in many centres. Cancellous bone can also be harvested from tibia and to a smaller extent from the mandible. Cranial bone and rib have also been used but tooth eruption through these grafts has not been demonstrated as successfully as with iliac and tibial bone. Also bone-inducing techniques have been demonstrated using pedicled periosteum or free tibial periosteal grafts with moderate results.

The source of the bone graft does not seem to primarily influence the success of the outcome. However, disagreement exists about different donor sites regarding the viability of autogenous bone, morbidity, amount of bone required, type of bone needed (cortical or cancellous), and expected biological behaviour (neovascularization and resorption). Furthermore, the procedure should aim at optimal physiological and psychological function causing minimal impairment of growth and development in the maxillo-facial complex.

Possible complications from the iliac crest may be excessive blood loss, haematoma, delayed wound healing, pain lasting for two weeks to two months, long, adherent and painful scars under belts or clothing and hypoesthesia or anaesthesia over the distribution of the lateral femoral cutaneous nerve. In the cranium there is a risk of penetration of the inner table and harvesting bone graft from the mandible which could cause damage to the roots of the canine and incisor and injury of the mental nerve. Harvesting rib grafts may sometimes lead to post-operative chest infection or pneumothorax

### Bone grafting in Gӧthenburg

**Figure 1:**
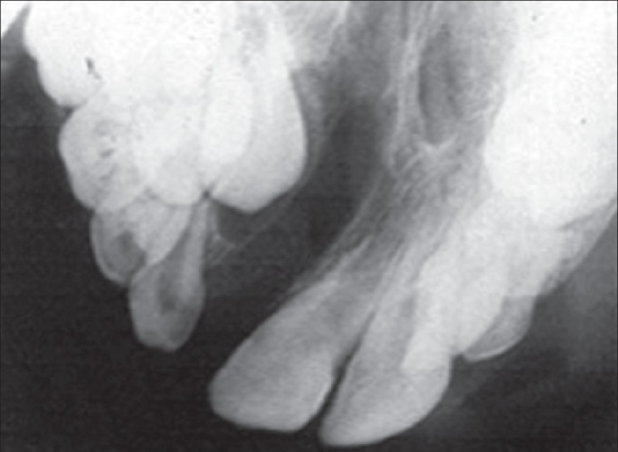
X-ray of the cleft area where the canine is still covered by a thin layer of bone. This situation when the canine has not yet erupted in to the cleft area is ideal for bone grafting

The optimal age for bone grafting is considered in most centres to be between the eighth and eleventh years when the root of the permanent canine has been formed about a quarter to two thirds its length. X-rays of the cleft area are taken at the age of about seven. The stage of eruption of the canine or lateral incisor, if present, can then be estimated. In Gothenburg we plan to bone graft the cleft when the tooth that is going to erupt in the cleft area (canine or lateral incisor) is still covered by a thin shell of bone. This is a little earlier than in most centres but in our unit it has increased the success rate of the grafts. [Figures 1 and 4]

**Figure 2A:**
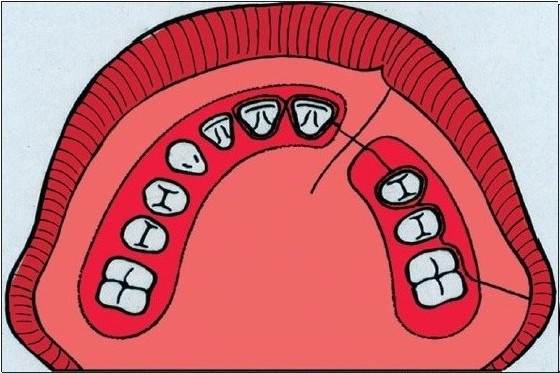
Schematic drawing of the bone grafting procedure. The incision lines along the gingival margin and the margin of the cleft

**Figure 2B:**
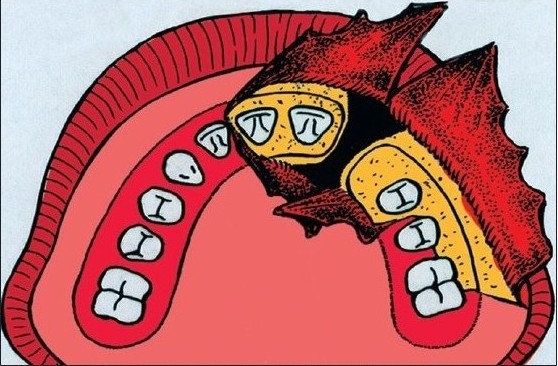
Gingival and palatal mucoperiosteal flaps raised

**Figure 2C:**
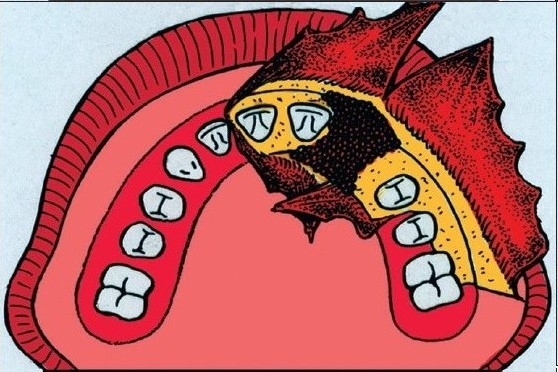
Bone grafting to the cleft in the alveolar process

**Figure 2D:**
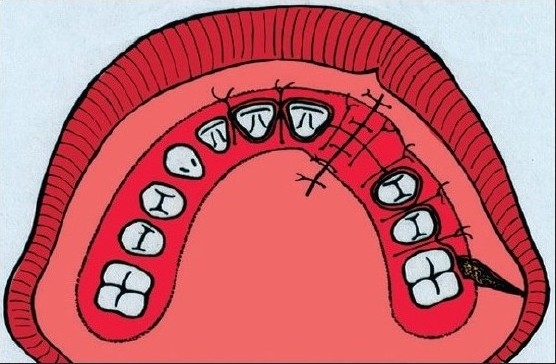
The bone graft covered with the palatal and gingival mucoperiosteal flaps. Note that the lateral gingival flap has been mobilised anteriorly to ensure good covering of the grafted area leaving the defect for secondary epithelialization in the region of the first molar.

**Figure 3A:**
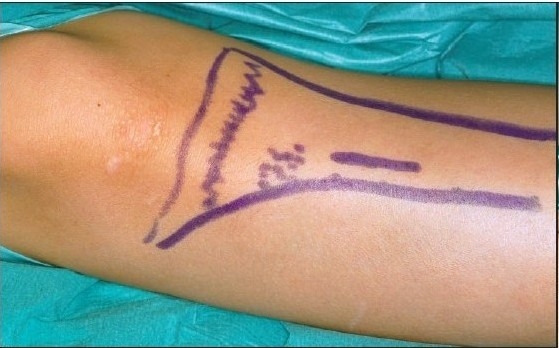
The bone grafts are harvested from the proximal part of the tibia just distal to the tuberosity. A) An incision about 10-15 mm is made in the proximal part of tibia immediately distal the to the tuberosity.

**Figure 3B:**
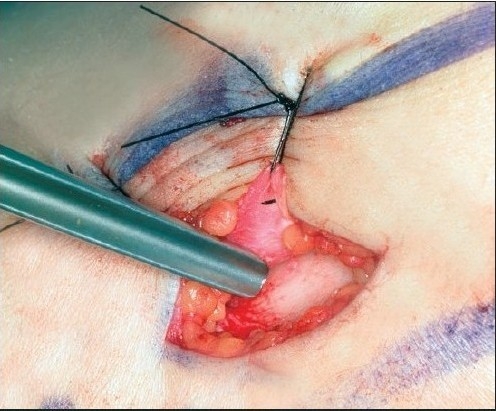
After incision a small periosteal flap is raised and pulled aside with sutures. A small window is then opened in the cortical wall with a chisel

**Figure 3C:**
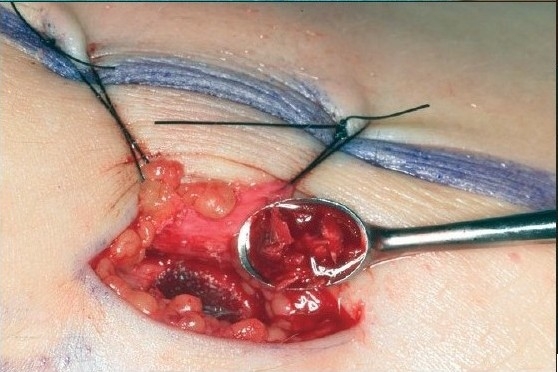
A cortical piece of the tibia bone is first harvested and put aside. With a curette cancellous bone is collected and put into a small bowl. Liquid from the marrow is aspirated and mixed with the cancellous bone graft in order to keep it moist. The defect in the tibia is covered with the periosteal flap, which is sutured back in place

**Figure 3D:**
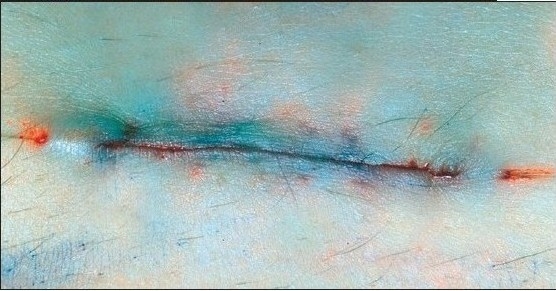
Subcuticular water tight closure by resorbable sutures in order to avoid oozing from the bone. The skin is closed by intradermal continuous suture

**Figure 4:**
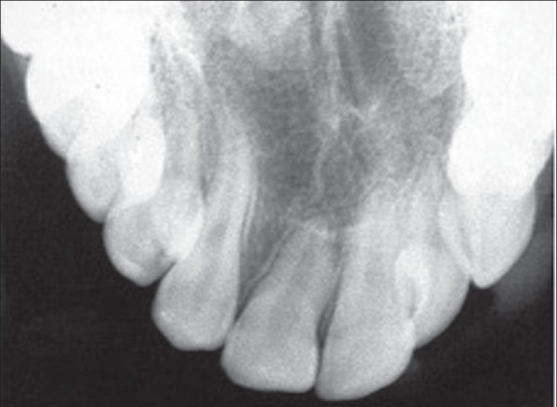
Post Alveolar Bone Graft Xray

In patients with UCLP, our main indication is to facilitate eruption of the lateral incisor and the canine in the cleft area. Augmentation of the alar base of the nose is not recorded as a major indication.

### Orthodontic treatment

When the stage of tooth eruption in the cleft area is clarified, orthodontic treatment is discussed. If the alveolar ridges are aligned and teeth are in good position, pre-operative orthodontic treatment is not needed. Sometimes the alveolar ridges block the entrance to the cleft area. Pre-operative orthodontic treatment can then be necessary to expand the arch and facilitate surgical accessibility. The central incisor close to the cleft usually has a very thin bony septum towards the cleft. This should be considered if orthodontic treatment is performed on these teeth. Deciduous teeth that may interfere with the bone grafting should be removed two to three months before bone grafting. It was found that extraction of deciduous teeth during the bone grafting procedure correlated to less remaining bone and to persistent gingival retraction. The harmful effect of extraction of a deciduous tooth in connection with bone grafting may be related to osteoresorptive cells and an environment suitable for bone resorption.

The success of bone grafting is also highly dependant on good oral hygiene. Teeth with carious lesions should be conservatively treated or extracted.

The Gӧthenburg technique for bone grafting procedure is described by Lilja *et al.* 1987. The labial incision lines were made along the gingival margins, on the cleft side to the first molar and on the non-cleft side to the canine. Two gingival flaps were then raised and a back-cut was made in the molar region. Palatal mucoperiosteal flaps were elevated enough to visualize the cleft up to the piriform aperture and the anterior nasal spine. This dissection enables trimming of palatal bone margins. In patients in whom the alar base was augmented, the dissection was widened. If a vestibular fistula was present, the nasal mucosa was mobilized and sutured. Cancellous bone, harvested from tibia was packed in the pocket created in the alveolar region. Finally, the gingival flaps were sutured to cover the bone graft. [Figure 2]

In a follow-up study our success rate for for bone grafting was found to be 97%. This is in accordance with other centres with well established protocols.

The aim in every cleft palate patient is to provide a complete dental arch without the need for a bridge or removable prostheses. If the lateral incisor can be saved, orthodontic space closure of the dental arch is easier to carry out.

This was confirmed in our patients in whom permanent lateral incisor was present in 71%. When bone grafting was done to facilitate eruption of the lateral incisor in our unit, space closure by orthodontic means was achieved in 100% of patients. When there were no lateral incisors present, the cleft gap in the dental arch could be closed orthodontically in only 81% of the patients.

The alveolar bony height has been an important variable to assess the success rate of grafting. In our patients, the results showed that 94% had normal or almost normal alveolar bony height. The mesial bony height was similar to that of distal bony height.[8]

Bone grafting when the erupting tooth is still covered by a thin shell of bone also achieved greater alveolar bony height than if it was done later. It may also prevent external root resorption which often can be found in patients bone grafted after canine eruption.

### Tibia as donor site for bone grafting

Since 1958 the tibial donor site has been used, in our department, providing minimal early and late morbidity. The harvesting time is a mere 15 minutes, there is negligible bleeding, and two teams can work simultaneously, reducing operative time and hospital costs and sufficient bone can be harvested with minimal peri-operative complications.

Furthermore, the short scar length and the minimal early and late morbidity make the choice of the donor site psychologically acceptable for children and parents. It might be argued that the scar is difficult to hide, but in our patients there were no complaints about this.[9]

The present technique for harvesting the bone grafts from tibia is described below:

Harvesting bone grafts from the tibia involves an incision about 10 mm long made in the proximal part of the tibia immediately distal to the tuberosity. After incision, a small periosteal flap is raised and pulled aside. A small window is then opened in the cortical wall with a chisel. Cancellous bone is harvested with a currette and put into a small cup where it is covered by blood or serum, not put into saline. Finally the graft is cut up with scissors and packed into the already prepared alveolar pocket in the cleft area. A satisfactory amount of cancellous bone could always be obtained. No drains are used. The periosteal flap is repositioned and sutured with absorbable suture materials. The skin is closed with an intracutaneous suture. A mild pressure dry dressing is then applied. Penicillin-derived antibiotics are given during the peri-operative period. Patients were mobilized on the day following the operation and could engage in normal activities and sports one month after surgery. The complications of harvesting bone grafts from tibia were few. There were no major fractures and minimal local problems occurred which could be handled in routine outpatient follow-up [Figure 3].

## CONCLUSIONS

To achieve optimal results, especially orthodontic space closure, bone grafting should be performed after eruption of the central incisors in the cleft region.

Bone grafting should be considered, when possible, to facilitate eruption of lateral incisors if present. If lateral incisors can erupt into the grafted bone, these teeth can be used, which will increase the possibilities for bony support and orthodontic space closure.

Surgical experience in performing the bone grafting procedure will give more favourable, long-term results. Therefore, it is recommended that only experienced surgeons should perform bone grafting.

As an indicator for timing of the bone grafting procedure, it is preferable to employ the remaining thickness of bone covering the crown (of the lateral incisor or of the canine), rather than use the degree of root formation of these teeth. The tibial donor site harvesting technique carries less early and late morbidity. This suggests that the tibia is an excellent choice to graft the residual cleft in cleft lip/ palate patients.
